# Characterizing Off-center MRI with ZTE

**DOI:** 10.1016/j.zemedi.2022.09.002

**Published:** 2022-10-31

**Authors:** Serhat Ilbey, Matthias Jung, Uzay Emir, Michael Bock, Ali Caglar Özen

**Affiliations:** a Dept. of Radiology, Medical Physics, Medical Center University of Freiburg, Faculty of Medicine, University of Freiburg, Freiburg, Germany; b Department of Diagnostic and Interventional Radiology, Medical Center University of Freiburg, Faculty of Medicine, University of Freiburg, Freiburg, Germany; c Weldon School of Biomedical Engineering, Purdue University, USA; d School of Health Science Department, Purdue University, USA

**Keywords:** Excitation profile, Off-center MRI, Short T2 imaging, Unwanted slice selection, Zero echo time, ZTE

## Abstract

**Purpose:**

To maximize acquisition bandwidth in zero echo time (ZTE) sequences, readout gradients are already switched on during the RF pulse, creating unwanted slice selectivity. The resulting image distortions are amplified especially when the anatomy of interest is not located at the isocenter. We aim to characterize off-center ZTE MRI of extremities such as the shoulder, knee, and hip, adjusting the carrier frequency of the RF pulse excitation for each TR.

**Methods:**

In ZTE MRI, radial encoding schemes are used, where the distorted slice profile due to the finite RF pulse length rotates with the k-space trajectory. To overcome these modulations for objects far away from the magnet isocenter, the frequency of the RF pulse is shifted for each gradient setting so that artifacts do not occur at a given off-center target position. The sharpness of the edges in the images were calculated and the ZTE acquisition with off-center excitation was compared to an acquisition with isocenter excitation both in phantom and *in vivo* off-center MRI of the shoulder, knee, and hip at 1.5 and 3T MRI systems.

**Results:**

Distortion and blurriness artifacts on the off-center MRI images of the phantom, *in vivo* shoulder, knee, and hip images were mitigated with off-center excitation without time or noise penalty, at no additional computational cost.

**Conclusion:**

The off-center excitation allows ZTE MRI of the shoulder, knee, and hip for high-bandwidth image acquisitions for clinical settings, where positioning at the isocenter is not possible.

## Introduction

Off-center field-of-view (FOV) MRI is used in many clinical applications where anatomic regions are located asymmetrically relative to the body axes, such as imaging of the shoulder, knee, and hip. In MRI with Cartesian k-space sampling, the in-plane off-center shift is implemented by adding a linear phase increase (Fourier shift theorem) prospectively during acquisition to the radio-frequency (RF) pulse and analog to digital converter to shift the off-center region into the center of the image. This approach can be generalized to arbitrary sampling schemes [1]. Alternatively, off-centered FOV can be applied after acquisition retrospectively during the image reconstruction by modulating the MR signal.

**Figure 1:**
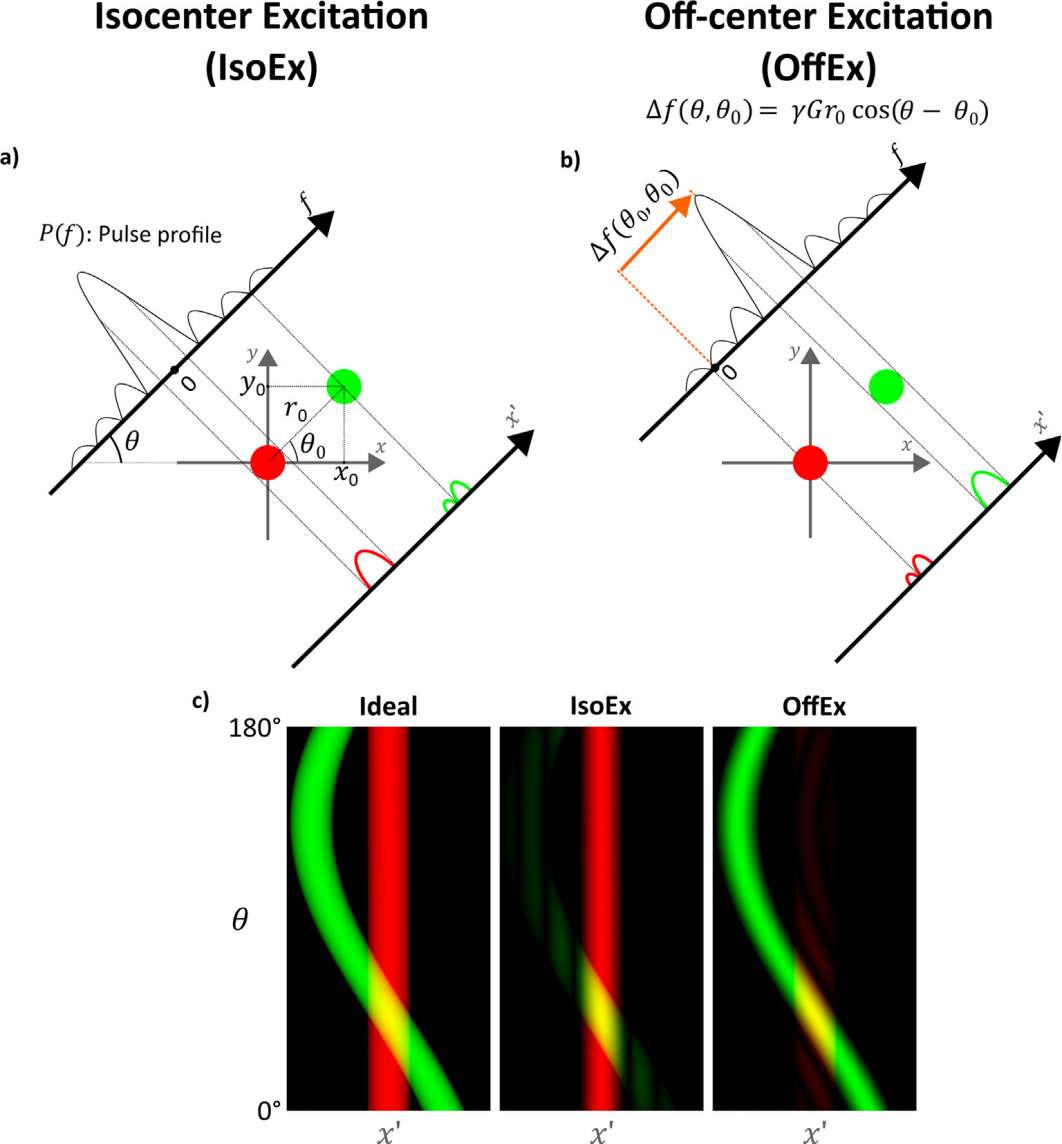
Illustration of the slice profile and its effect in 2D. Projections of 2D numerical phantoms with isocenter excitation (IsoEx) **(a)** and the off-center excitation (OffEx) **(b)** are presented. The phantom in red is positioned at the isocenter, i.e., at $\left(x,y\right)=\left(0,0\right)$, whereas the phantom in green was positioned at $\left(x,y\right)=\left(x_{0},y_{0}\right)$, where $r_{0}=\sqrt{x_{0}^{2}+y_{0}^{2}}$ and $\tan^{-1}\left(\frac{y_{0}}{x_{0}}\right)=\theta_{0}$. For the given gradient angle θ, the projection of the object at the off-centered position is highly distorted with IsoEx, whereas it was corrected by shifting the slice profile **(b)**. For each projection angle, $\Delta {f(\theta ,\theta }_{0})$ must be recalculated using the given formula. In **(c)** sinograms of the same numerical phantoms were presented for ideal excitation, IsoEx, and OffEx, respectively. The artifacts due to pulse profile were mitigated for the object at the off-centered position when OffEx is used, at the expense of introducing artifacts to the object at isocenter. As the slice profile is still not flat when the carrier frequency of the RF pulse is shifted with OffEx, projections were still modulated with the shifted profile, e.g., they look thinner than the ideal projections.

Sequences with ultra-short TE, such as UTE [2], ZTE [3], [4], [5], [6], [7], [8], and SWIFT [9], or even with a truly simultaneous acquisition and excitation [10], [11] have been developed to image tissues with ultra-short T_2_ such as bones [12], [13], tendons [14], teeth [15], [16], and for MRI of X-nuclei [17], [18]. In ZTE frequency-encoding gradients are already switched on before the application of the short RF pulse for excitation to shorten TE; however, the presence of a gradient during RF excitation leads to an unwanted spatial selectivity (Fig. 1). Unlike conventional 3D pulse sequences, the slice selectivity during the RF pulse rotates with the k-space trajectory. The excitation bandwidth also changes as the gradient strength depends on the acquired k-space sample. For example, an 8µs-long rectangular RF pulse applied in a 20 mT/m gradient field results in a sinc-shaped slice profile along the gradient direction with a full-width-half-maximum (FWHM) of 17.5 cm. This leads to blurring at the edge of the FOV, which cannot be recovered during post-processing as for some positions in the image the unwanted slice-selection leads to a zero-crossing of the excitation profile resulting in a non-ideal point spread function.

To mitigate slice profile artifacts, RF pulses can be shortened using dedicated hardware [6] and gradient amplitudes can be reduced during RF excitation [19], [20]. The effect of the signal can also be partly removed during post-processing [21], [22], which requires measurement of the slice profile and adds computational complexity. However, these methods do not address the artifacts in off-centered objects, where the distortions on the excitation profile are even stronger. So far, the only solution to minimize the artifacts is to place the region of interest as close as possible to the isocenter, which decreases the patient comfort or might be impossible for oversized patients or patients with limited motion capabilities. In some studies ZTE MRI of off-centered body parts such as shoulder and hips have already been demonstrated [23], [24]. However, the effect of the off-centered excitation on the ZTE images was not mentioned. In [6], this problem was mentioned and related to the frequency of the RF pulse in ZTE, yet, its applications and the effect on the image quality have not been demonstrated.

In this study, we characterize and analyze the RF frequency modulation scheme for off-centered objects in which the carrier frequency of the RF pulse is shifted such that the center of the RF pulse profile coincides with the centroid of the object, i.e. point of interest (POI), for each projection angle. The frequency shift is calculated from the distance between the POI and the isocenter, gradient amplitude, and the projection angle. This per-shot carrier-frequency-adjusted off-center excitation (OffEx) scheme [6], [25] was compared to the fixed-carrier-frequency excitation, hereinafter referred to as Isocenter Excitation (IsoEx), in both phantom and *in vivo* measurements, which were performed in extremities such as the shoulder, knee, and hip at 1.5 and 3T using a ZTE sequence [26].

## Methods

The carrier frequency offset Δf of the RF pulse for an off-centered excitation is given by

(1) $$\Delta f\;=\gamma \left(G\cdot r_{0}\right)=\gamma \left(G_{x}x_{0}+G_{y}y_{0}+G_{z}z_{0}\right)$$

(2) $$=\gamma Gr_{0}\left(\cos\left(\theta -\theta_{0}\right)\sin\phi \sin\phi_{0}+\cos\phi \cos\phi_{0}\right)$$

where γ is the gyromagnetic ratio, the gradient vector $G=\left(G_{x},G_{y},G_{z}\right)$ during RF excitation, and the off-center position $r_{0}=\left(x_{0},y_{0},z_{0}\right)$. θ and ϕ represent the azimuthal and polar angles of the readout gradients in k-space, respectively, whereas $\theta_{0}$ and $\phi_{0}$ represent the position of the point object in image-space (see Appendix A). In Eq. 1 Δf depends not only on the gradient amplitude $\left|G\right|=G$ and ${|r}_{0}|=r_{0}$, but also on the direction of the applied gradient during excitation and the position of the object. Using this OffEx scheme, artifacts due to slice profile at $r_{0}$ are fully removed (Fig. 1). However, as the slice profile is still not flat when the carrier frequency of the RF pulse is shifted adaptively, signals from positions away from $r_{0}$ are still modulated with the shifted profile. As seen from the sinograms presented in Fig. 1c, artifacts due to pulse profile were mitigated for the object at the off-centered position when OffEx is used, at the expense of introducing artifacts to the object at isocenter.

**Figure 2:**
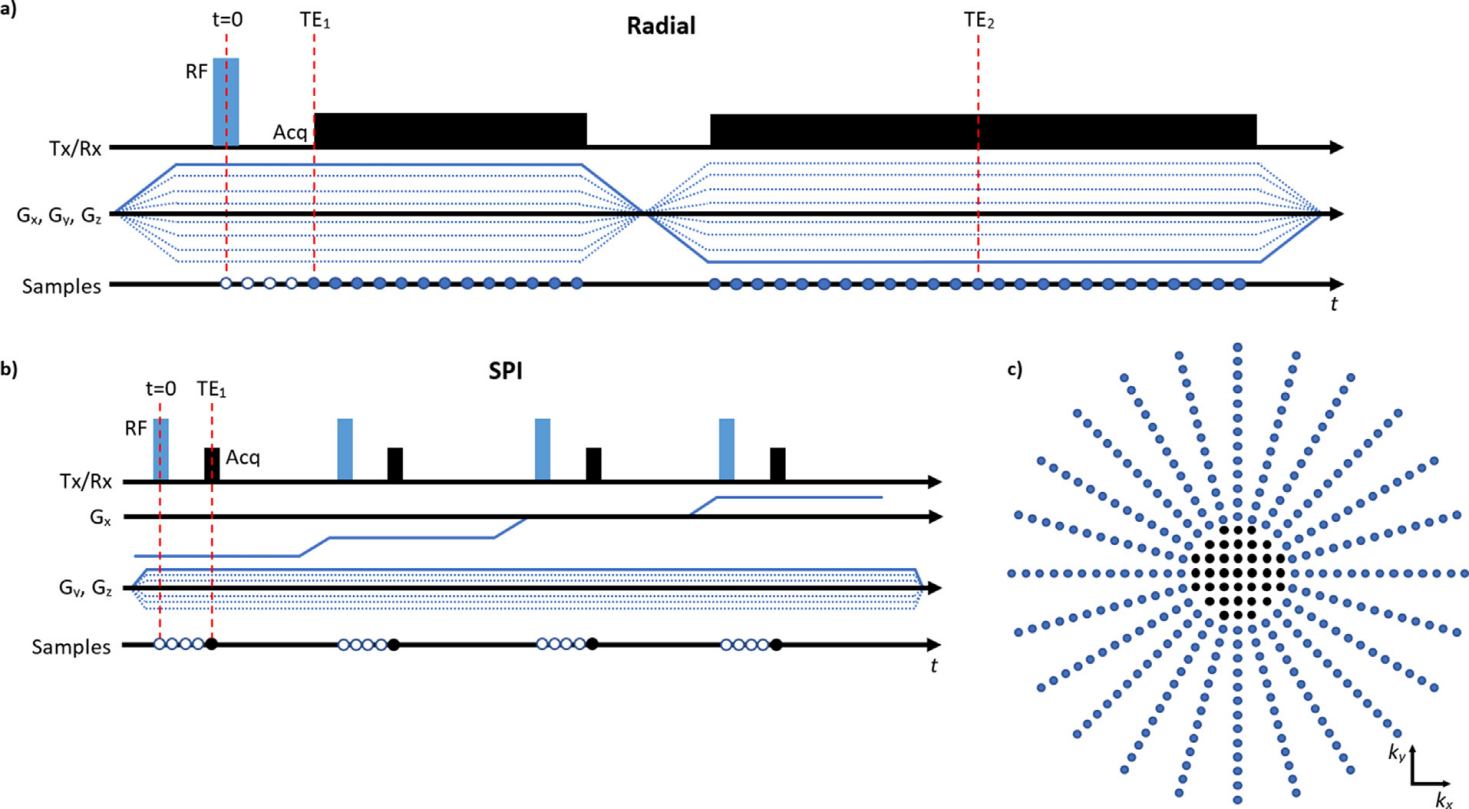
ZTE sequence diagram. **a)** Diagram of the radial acquisition. Gradients are already ramped-up before the hard pulse excitation. Gradient amplitude is constant, and only its direction is changed for every sequence block. To acquire a second echo, the polarity of the frequency-encoding gradients were inverted after acquiring the first echo. For spoiling, gradients are ramped directly to the strength required for the next repetition. **b)** Diagram of the SPI acquisition. Gradients are stepped through to acquire the k-space region, which cannot be acquired with the radial part. **c)** A 2D example of positions of the samples in k-space acquired with radial acquisition (blue) and SPI method (black).

The OffEx method was implemented in a ZTE sequence (Fig. 2) which combines radial spokes for the outer parts of k-space with Single Point Imaging (SPI) at the center [7]. With SPI, data at the spherical gap at the k-space center due to the dead time between the excitation and acquisition is recovered. SPI samples were collected in Cartesian coordinates at t=TE with gradient $G=k/\left(\gamma TE\right)$. In the radial part, G was kept constant but its direction is changed at every TR to create radial spokes that fill a spherical shell with equidistant points [7]. The POI $\left(x_{0},y_{0},z_{0}\right)$ is defined by the user and is then retrieved from the graphical user interface of the MR system. At each TR, Δf is re-calculated for the given gradient amplitude and direction.

Phantom and *in vivo* measurements (one 32-year-old male healthy volunteer) were performed to evaluate the imaging performance for various off-center shifts up to 17 cm. MRI measurements were performed at a 1.5T clinical system (MAGNETOM Aera, Siemens AG, Erlangen, Germany) and at a 3T (MAGNETOM Prisma Fit, Siemens AG). ZTE images of a resolution phantom were acquired using a receive-only loop coil (Ø = 11 cm, Siemens AG). Images of the left shoulder and the right knee of the healthy volunteer were acquired at 1.5T with a 16-channel receive-only small shoulder coil (Siemens AG), and 15-channel transmit/receive knee coil (QED, Cleveland, OH), respectively. Images of the right hip of the healthy volunteer were acquired at 3T with an 18-channel receive-only body matrix (Siemens AG). For reference, T_1_-weighted TSE images were acquired. Detailed parameters are listed in Supporting Information Table S1.

ZTE images were acquired with the following parameters for shoulder/knee/hip: FOV = (180 mm)^3^, voxel size = (0.5 mm)^3^, TE = 0.05 ms, τ =  8 µs, α = 3°/6°/3°, G = 20 and 30 mT/m. To acquire a second echo, the polarity of the frequency-encoding gradient was inverted after the acquisition of the first echo. The 3D radial spokes were ordered following a spiral pattern [27]. All acquisitions for single / double echo protocols are highly under-sampled (acceleration rates of 3 / 5) in the azimuthal direction to limit the acquisition time to 5 / 8 min.

The POI of the shoulder, knee, and hip was $\left(x_{0},y_{0},z_{0}\right)$ = (10,0,0), (8.5,0,0), and (10,3,1) cm, respectively. The phantom images were acquired at the isocenter (0,0,0), and at various off-centered positions, namely (10,0,0), (0,-10,0), (10,-10,0), and (10,-10,10). For τ = 8 µs and G = 20 and 30 mT/m, the first zero crossing point ($r_{\mathrm{zc}}$) was at 14.7 and 9.8 cm, respectively. Hence, severe artifacts were expected especially for G = 30 mT/m.

To reconstruct ZTE images radial samples were first re-gridded onto a Cartesian grid and then combined with the SPI samples after applying a density compensation filter. Next, a Hamming filter is applied to reduce ringing artifacts and noise [28], [29]. Afterwards, k-space data is modulated to shift the image to the off-center position. Finally, 3D Fourier transform of the k-space data is calculated.

A 2D example of the acquired signal and the reconstructed projections of the resolution phantom at $\left(x_{0},y_{0},z_{0}\right)$ = (10,0,0) are presented for projections at z=0 for IsoEx and OffEx in Supporting Information Video S1.

The reconstruction of a 3D data set took approximately 5 minute per channel using a workstation with a 3.6-GHz six-core CPU, NVIDIA GeForce RTX 3090 GPU, and 128 GB RAM. GPU was used only for re-gridding.

## Data analysis

To visualize the extent of geometric distortions and blurring artifacts, absolute difference images were calculated. The quality of the images acquired with IsoEx and OffEx was evaluated quantitatively by assessing the edge sharpness. Hence, line profiles were selected from the regions of interest where the highest distortions were expected. Afterwards, the Signal Intensities (SI) along the line profiles were fitted to a sigmoid function:

(3) $$\text{S}\text{I}\left(i;c_{0},c_{1},c_{2},s\right)=\frac{c_{1}}{1+10^{s\left(c_{0}-i\right)}}+c_{2}$$

where i is the independent variable representing the pixels along the line profile, and $c_{0}$, $c_{1}$, and $c_{2}$ determines the center location, vertical range, and the vertical offset, respectively. s is the parameter which defines the sharpness of the sigmoid and, hence, the line profile [30].

### Implementation

The OffEx method can be implemented in various ways depending on the pulse sequence programming environment. RF pulse and the data acquisition events need to be in phase, so the phase of the frequency-shifted RF pulse must be set for each frequency shift correctly. We implemented the ZTE sequence at the MRI systems with syngo MR C/E11 software version, where the minimum duration between frequency/phase events was hard-coded as 20 µs. As the pulse duration was 8 µs the numeric controlled oscillator (NCO) phase was set and reset (20–8)/2 = 6 µs before the start of the pulse and 6 µs after the end of the pulse. Instead, one can generate a 20 µs-long arbitrary shaped hard pulse but with zero amplitude at the first and last 6 µs of the generated pulse. Moreover, the actual RF pulse shape deviates from the ideal amplitude modulation due to the finite ramp up and ramp down times of the RF power amplifiers. We measured the pulse shape using a small pick-up loop coil and determined an asymmetry factor of 0.53, compared to the ideal rectangular pulse. This value was used to correctly set the NCO phase.

## Results

**Figure 3:**
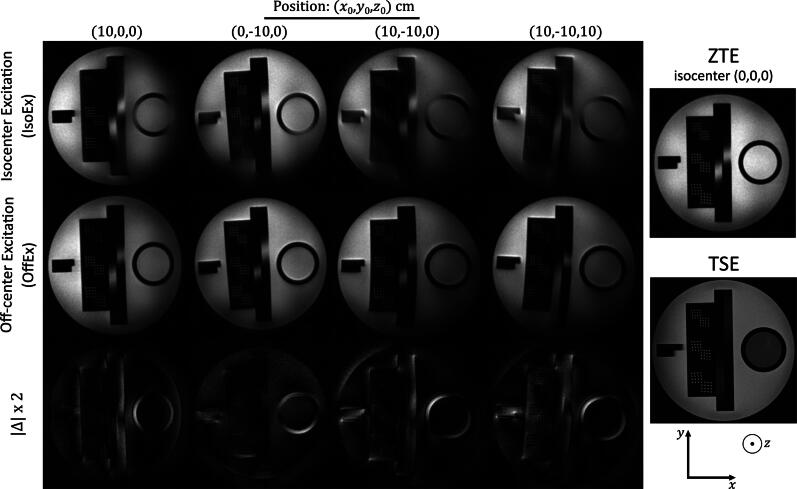
Phantom images acquired with G = 20 mT/m and acquired at isocenter and various off-centered positions (10,0,0), (0,-10,0), (10,-10,0), and (10,-10,10) using IsoEx and OffEx are shown together with the twice up-scaled absolute difference images ($\left|\Delta \right|\;x\;2$). A TSE image is presented for reference. The severity of the artifacts increased with increasing distance to the isocenter. The OffEx method mitigates the distortion and blurring artifacts at the images of the off-centered objects.

In Fig. 3, phantom images acquired with G = 20 mT/m using IsoEx and OffEx are shown together with the twice up-scaled absolute difference images. A TSE image is presented for reference. The severity of the artifacts increased with increasing distance to the isocenter. The OffEx method mitigates the distortion and blurring artifacts at the images of the off-centered objects. With OffEx, the acquired image had a similar image quality compared to the one acquired at the isocenter. Using higher gradient amplitudes (i.e., higher bandwidth per voxel) resulted in more severe artifacts (Supporting Information Fig. S1). Stronger geometric distortion artifacts due to the gradient nonlinearities were observed for the objects further away from the isocenter (e.g., for the phantom at $\left(x_{0},y_{0},z_{0}\right)$= (10,-10,10)).

Calculated sharpness (s) of the selected edge of the phantom positioned at $\left(x_{0},y_{0},z_{0}\right)$ = (10,0,0) and isocenter are presented at Supporting Information Fig. S2. The median of the sharpness of the edges for the object at isocenter (Obj-Iso) and at $\left(x_{0},y_{0},z_{0}\right)$ = (10,0,0) with OffEx and IsoEx acquired with G = 20 mT/m were 1.0, 1.1, and 0.38, respectively. Off-centered phantom images acquired with IsoEx had severe blurring artifacts, whereas the edges of the phantom were fully preserved with OffEx. Similar results were obtained for the images acquired with G = 30 mT/m.

**Figure 4:**
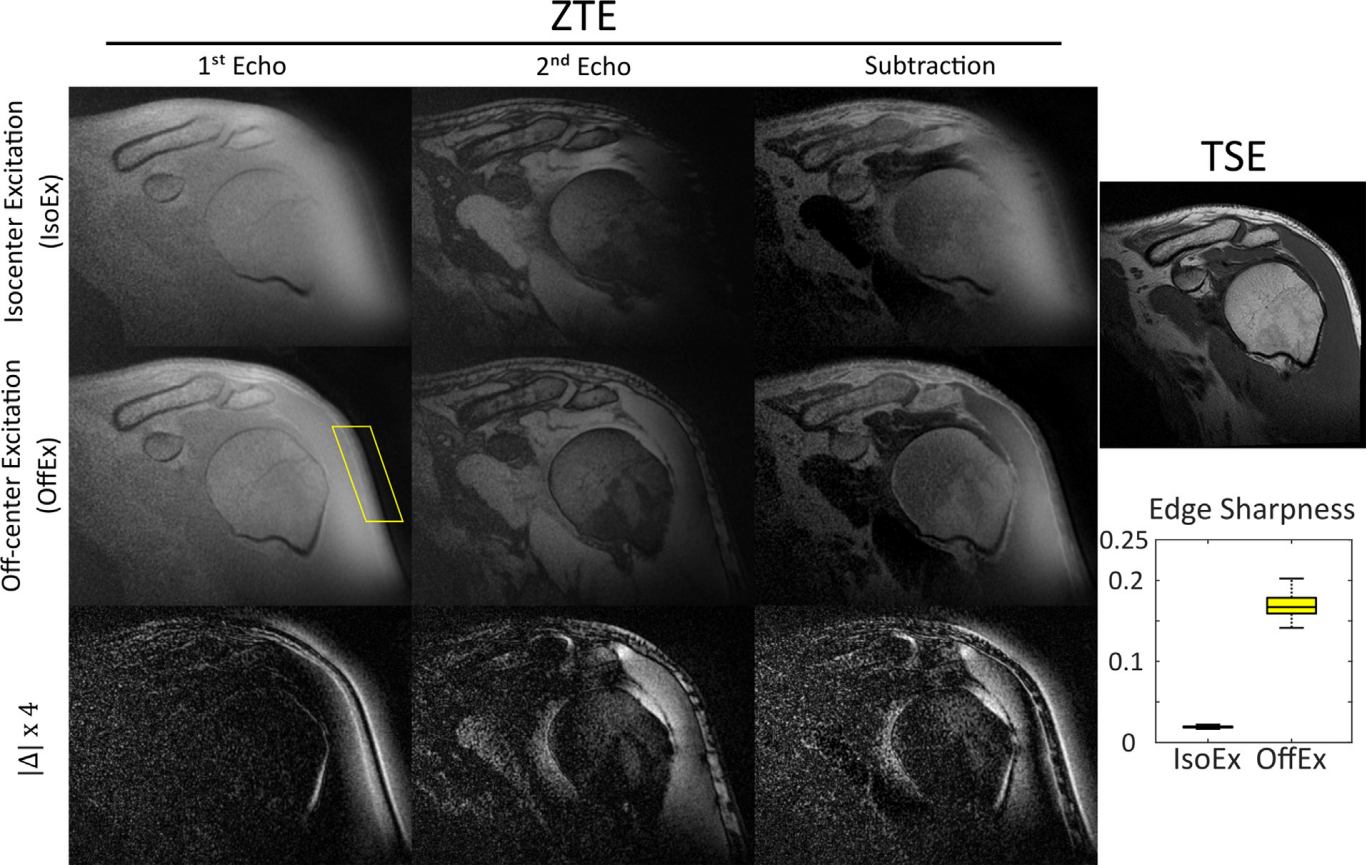
Left shoulder images of the healthy volunteer positioned at $\left(x_{0},y_{0},z_{0}\right)$ = (10,0,0) acquired with double-echo ZTE sequence with G = 20 mT/m using IsoEx and OffEx are presented together with the four times up-scaled absolute difference images ($\left|\Delta \right|\;x\;4$). A conventional T_1_-weighted TSE image is shown for reference. OffEx mitigates the distortion and blurring artifacts of the images. The sharpness of the selected surrounding fat tissue was increased from 0.02 to 0.17, when OffEx is used. The SNR of the deltoid muscle in the 2^nd^ echo was 9.9 and 25.9 for IsoEx and OffEx, respectively.

In Fig. 4, left shoulder images of the healthy volunteer acquired with a double-echo ZTE sequence with G = 20 mT/m using IsoEx and OffEx are presented together with the four times up-scaled absolute difference images and a conventional T_1_-weighted TSE image. With the increasing distance to the isocenter, the amount of distortions and blurring increases. In 1^st^ echo, the lateral aspect of the shoulder, including the humeral head acromion, deltoid muscle, and the subcutaneous fat are severely blurred and distorted as expected from the sinc-shaped excitation profile. The use of OffEx significantly improved the visualization of lateral bony structures such as the humerus and acromion; in particular, bone marrow and cortical bone are clearly delineated and there is a distinct contrast between the musculature and the bone. The median of the edge sharpness calculated at the surrounding fat region, at which the biggest distortions were visually observed, was increased from 0.02 to 0.17 when OffEx is used. The hyperintense signal artifact at the deltoid muscle in the conventional ZTE images was reduced and the dark band in the 2^nd^ echo was also recovered. The SNR of the deltoid muscle in the 2^nd^ echo was 9.9 and 25.9 for IsoEx and OffEx, respectively.

**Figure 5:**
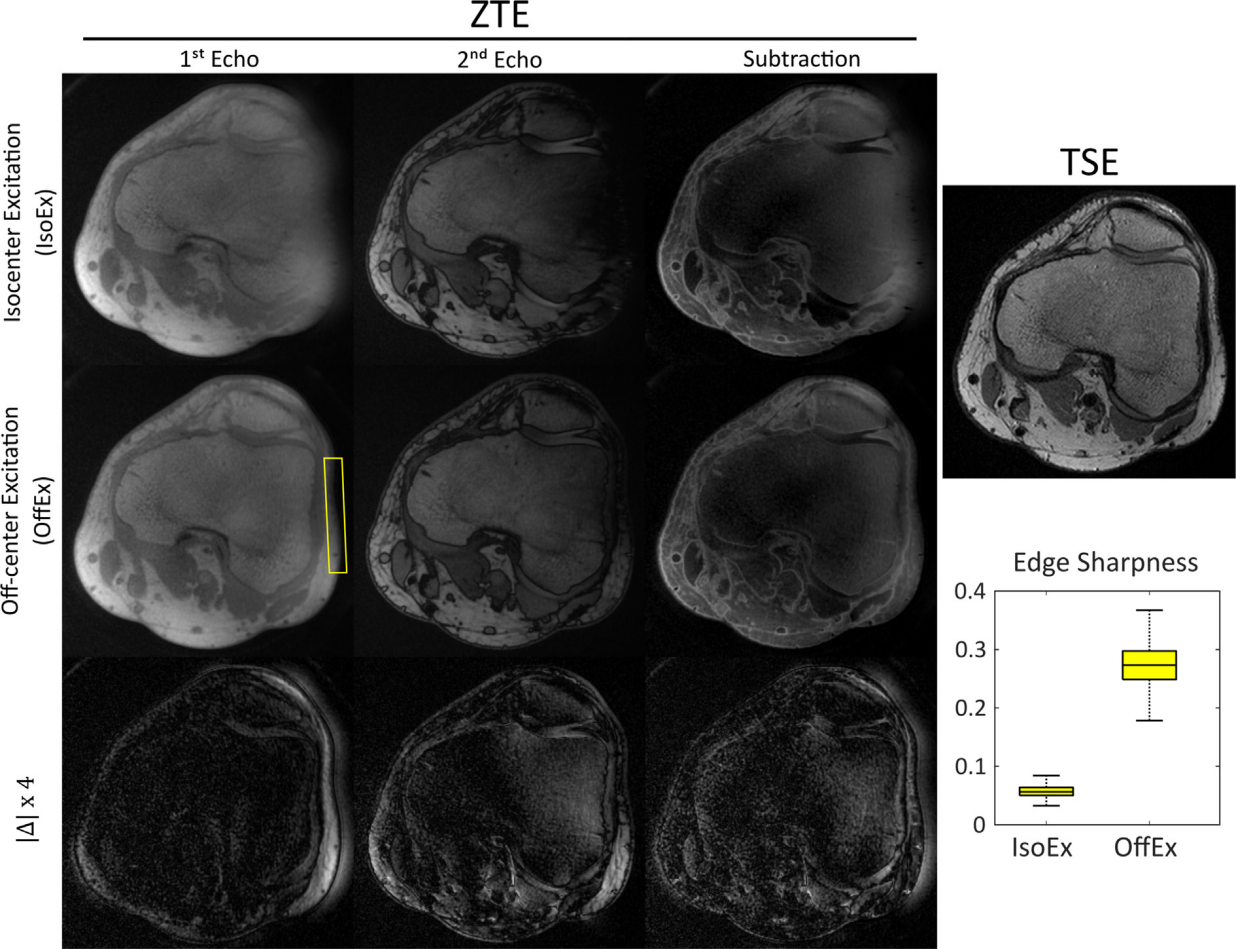
Right knee images of the healthy volunteer positioned at $\left(x_{0},y_{0},z_{0}\right)$ = (8.5,0,0) acquired with double-echo ZTE sequence with G = 20 mT/m using IsoEx and OffEx are presented together with the four times up-scaled absolute difference images ($\left|\Delta \right|\;x\;4$). A conventional T_1_-weighted TSE image is shown for reference. OffEx mitigates the distortion and blurring artifacts of the images. The sharpness of the selected surrounding fat tissue was increased from 0.06 to 0.27, when OffEx is used. The SNR of the periarticular musculature in the 2^nd^ echo was 26.5 and 48.8 for IsoEx and OffEx, respectively.

In Fig. 5, double-echo ZTE images of the right knee of the healthy volunteer are shown with G = 20 mT/m using IsoEx and OffEx methods. In the 1^st^ echo of the IsoEx ZTE data, the patella, and the lateral compartment of the knee including the lateral femoral condyle, biceps femoris and plantaris muscle, and the subcutaneous fat are blurred. OffEx recovered the distorted parts of the patella and the lateral knee compartment. The median of the edge sharpness was increased from 0.06 to 0.27 when OffEx is used. Similar to the shoulder, the cortical bone can now be delineated from the bone marrow and the contrast between bone, cartilage and the periarticular musculature, and fat is enhanced. The SNR of the periarticular musculature in the 2^nd^ echo was 26.5 and 48.8 for IsoEx and OffEx, respectively.

In Supporting Information Figs. S3 and S4, left shoulder and right knee images of the volunteer are presented for G = 30 mT/m, respectively. Supporting Information Fig. S5 shows the right hip images of the healthy volunteer positioned at $\left(x_{0},y_{0},z_{0}\right)$ = (10,3,1) acquired with ZTE sequence with G = 20 and 30 mT/m using IsoEx and OffEx. In the IsoEx images femur, m. gluteus medius, and the surrounding fat was highly blurry and distorted. For G = 20 mT/m the median of the edge sharpness was increased from 0.04 to 0.27 when OffEx is used, whereas for G = 30 mT/m it was increased from 0.03 to 0.14. Raw image data from the phantom and *in vivo* experiments are made available at https://github.com/serhatilbey/offcenterZTE.

## Discussion

In this work, OffEx was described and analyzed to characterize the image artifacts due to the unwanted slice selection in ZTE MRI of the body parts which cannot be positioned at the isocenter. Off-center imaging is often required for musculoskeletal applications such as hip, shoulder or knee imaging, and distances to the isocenter are especially large in oversized or adipose patients. ZTE is a promising technique that may allow accurate imaging of bone using MRI and could become a radiation-free alternative to computed tomography (CT) [31], [32], [33]. With the off-center excitation, the distortion and blurring artifacts on unilateral ZTE MR images of the shoulder, knee, and hip were significantly reduced. The contrast between bony structures and the periarticular soft tissues was markedly enhanced. Further, a clear delineation between the bone marrow and the cortical bone was achieved, which is crucial for clinical evaluation of bone and has so far been the strength of computed tomography compared to MRI. Moreover, with OffEx, these body parts do not have to be placed at the isocenter which increases patient comfort, especially for immobile patients with severe disease in the organ of interest.

The analyzed method, OffEx, can also overcome the flip angle limitations of the hard pulses in clinical MRI systems by using longer RF pulses. For example, if a specific organ is located away from the isocenter, longer hard pulses cause significant artifacts. However, with OffEx, the main lobe of the distorted excitation profile can be shifted towards the region of interest. Longer pulses may be played out to increase SNR at the expense of slight distortions in the surrounding anatomy.

OffEx using the rectangular RF pulses results in shifting the peak of the sinc excitation profile in space. Artifacts due to unwanted slice profile were still present in the images acquired with OffEx because the slice profile was still not flat for the FOV. Frequency modulated RF pulses [22] and gradient modulation techniques [19] to increase the excitation bandwidth can be combined with OffEx without loss of generality for ZTE MRI of body parts located away from the isocenter.

Combining the retrospective correction techniques [21], [22] with OffEx can further mitigate the distortion and blurriness artifacts. The technique proposed in [21] by Grodzki et al. enhances image quality as long as the first minimum of the excitation profile lies outside the imaged region. However, combining that retrospective correction method with OffEx can overcome this limitation.

In this work, data from each channel was reconstructed independently due to high memory requirements. However, reconstructing all channels using parallel imaging techniques together with the calculated coil sensitivity profiles might increase the overall image quality, which can be highly valuable in clinical practice.

## Conclusion

We demonstrated that with OffEx, high resolution and high bandwidth ZTE MRI is feasible for body parts that cannot be positioned at the isocenter. A significant reduction in image distortions was observed in unilateral ZTE MRI of the shoulder, knee, and hip for RF pulse duration of 8 µs at an isotropic resolution of 0.5 mm in 5 / 8 for min single / double echo protocols. This method can be implemented without additional hardware or post-processing. After minor modifications of the sequence source code, it could be easily integrated to existing protocols and clinical routine by adjusting the center of FOV to the position of interest via the standard user interface.

## Ethical approval

All methods were carried out in accordance with relevant guidelines and regulations, healthy volunteer scanning was approved by the Institutional Review Board (Ethikkommission) of the University Medical Center Freiburg (No. 160/2000), and informed written consent was obtained before imaging.

## Informed consent to participate

Informed consent was obtained from all individual participants included in the study.

## Informed consent to publish

Informed consent was obtained from all individual participants included in the study.

## Declaration of Competing Interest

The authors declare that they have no known competing financial interests or personal relationships that could have appeared to influence the work reported in this paper.

## Appendix A

In ZTE typically rectangular excitation pulses are used. The amplitude of these pulses is related to the pulse duration τ and the flip angle α via $B_{1}=\alpha /2\pi \gamma \tau$. According to the small-tip-angle approximation with $\alpha <30^{{}^{\circ}}$ [34] the normalized frequency response of a rectangular pulse is given by its Fourier transform:

(A.1) $$P\left(f\right)=\mathrm{sinc}\left(\pi f\tau \right)$$

When the gradients are already ramped up before the rectangular pulse is applied, this frequency response leads to a non-ideal excitation which is spatially modulated by the sinc profile:

(A.2) $$s\left(k\right)=\underset{V}{\overset{\;}{∭}}P\left(f\right)m\left(r\right)e^{-i2\pi (k\cdot r)}dr$$

where

(A.3) $$k\left(t\right)=\gamma \int_{0}^{t}G\left(t^{\prime }\right)dt^{\prime }$$

and

(A.4) $$f=\gamma \left(G\cdot r\right)$$

The frequency, f, is a function of the gyromagnetic ratio γ, the gradient vector $G=\left(G_{x},G_{y},G_{z}\right)$ during RF excitation, $G=\sqrt{G_{x}^{2}+G_{y}^{2}+G_{z}^{2}}$, and r=(x,y,z), which is the position in image-space. Substituting Eq. A.4 into Eq. A.1 yields

(A.5) $$P\left(G,r\right)=\mathrm{sinc}\left(\pi \gamma (G\cdot r)\tau \right)$$

As P is a function of both G and r, it leads to different amplitude modulations of the k-space signal for different gradient amplitudes and directions, which causes position-dependent blurring artifacts in the reconstructed images. The artifact severity increases with the distance from the isocenter because as r increases, P gets sharper (see Eq. A.5 and Fig. 1). Note that the excitation vanishes at the zero crossings of the pulse profile in image space, and the first zero crossing radius of the sinc function (Eq. A.5) is at $r_{\mathrm{zc}}={\left(\gamma G\tau \right)}^{-1}$.

The 3D point spread function (PSF) of an idealized MR system for a point object at position $r_{0}=\left(x_{0},y_{0},z_{0}\right)$ is defined as:

$$s\left(k_{x},k_{y},k_{z}\right)=∭P\left(G,r\right)∙\delta \left(x_{0},y_{0},z_{0}\right)∙e^{-i2\pi \left(k_{x}x+k_{y}y+k_{z}z\right)}dxdydz$$

(A.6) $$=P\left(G,r_{0}\right)∙e^{-i2\pi \left(k_{x}x_{0}+k_{y}y_{0}+k_{z}z_{0}\right)}$$

where $\left|r_{0}\right|=r_{0}=\sqrt{x_{0}^{2}+y_{0}^{2}+z_{0}^{2}}$ is the radial distance of the point object to the isocenter, $x_{0}=r_{0}\cos\theta_{0}\sin\phi_{0}$, $y_{0}=r_{0}\sin\theta_{0}\sin\phi_{0}$, $z_{0}=r_{0}\cos\phi_{0}$, $G_{x}=G\cos\theta \sin\phi$, $G_{y}=G\sin\theta \sin\phi$, and $G_{z}=G\cos\phi$. Here, θ and ϕ represent the azimuthal and polar angles of the readout gradients in k-space, respectively, whereas $\theta_{0}$ and $\phi_{0}$ represent the position of the point object in image-space. To mitigate the modulation of the slice profile $P\left(G,r\right)$ at position $r_{0}$, we shift the resonance frequency of the RF pulse by $\Delta {f(\theta ,\theta }_{0})$, so that the maximum of the sinc function is located at $r_{0}$:

(A.7) $$P\left(G,r_{0}\right)=\mathrm{sinc}\left(\pi \left[\gamma \left(G\cdot r_{0}\right)-\Delta {f(\theta ,\theta }_{0},\phi ,\phi_{0})\;\right]\tau \right)=1$$

Thus, for each acquisition the MR signal from $r_{0}$ is acquired with the same intensity, and the slice profile effect is removed. To fulfil Eq. A.7, the argument of the sinc function must vanish so that

(A.8) $$\Delta {f(\theta ,\theta }_{0},\phi ,\phi_{0})\;=\gamma \left(G\cdot r_{0}\right)=\gamma \left(G_{x}x_{0}+G_{y}y_{0}+G_{z}z_{0}\right)$$

(A.9) $$=\gamma \left(G\cos\theta \sin\phi r_{0}\cos\theta_{0}\sin\phi_{0}+G\sin\theta \sin\phi r_{0}\sin\theta_{0}\sin\phi_{0}+G\cos\phi r_{0}\cos\phi_{0}\right)$$

(A.10) $$=\gamma Gr_{0}\left(\cos\left(\theta -\theta_{0}\right)\sin\phi \sin\phi_{0}+\cos\phi \cos\phi_{0}\right)$$

For an object centered at $z_{0}=0$ in z-direction (hence, $\phi_{0}=90^{{}^{\circ}}$), Eq. A.10 reduces to

(A.11) $$\Delta {f(\theta ,\theta }_{0},\phi )\;=\gamma Gr_{0}\cos\left(\theta -\theta_{0}\right)\sin\phi$$

## References

[1] Magland J., Wehrli F. (2006). General algorithm for automated off-center MRI. Magn Reson Med Off J Int Soc Magn Reson Med.

[2] Robson M.D., Gatehouse P.D., Bydder M. (2003). Magnetic resonance: an introduction to ultrashort TE (UTE) imaging. J Comput Assist Tomogr.

[3] Lauterbur P.C. (1973). Image formation by induced local interactions: examples employing nuclear magnetic resonance. Nature.

[4] Hafner S. (1994). Fast imaging in liquids and solids with the Back-projection Low Angle ShoT (BLAST) technique. Magn Reson Imaging.

[5] Madio D.P., Lowe I.J. (1995). Ultra-fast imaging using low flip angles and FIDs. Magn Reson Med.

[6] Weiger M., Pruessmann K.P., Harris R.K. (2012). Hrsg. Encyclopedia of Magnetic Resonance.

[7] Grodzki D.M., Jakob P.M., Heismann B. (2012). Ultrashort echo time imaging using pointwise encoding time reduction with radial acquisition (PETRA). Magn Reson Med.

[8] Froidevaux R., Weiger M., Rösler M.B. (2021). HYFI: Hybrid filling of the dead-time gap for faster zero echo time imaging. NMR Biomed.

[9] Idiyatullin D., Corum C., Park J.-Y. (2006). Fast and quiet MRI using a swept radiofrequency. J Magn Reson.

[10] Idiyatullin D., Suddarth S., Corum C.A. (2012). Continuous SWIFT. J Magn Reson.

[11] Özen A.C., Atalar E., Korvink J.G. (2018). In vivo MRI with Concurrent Excitation and Acquisition using Automated Active Analog Cancellation. Sci Rep.

[12] Du J., Bydder G.M. (2013). Qualitative and quantitative ultrashort-TE MRI of cortical bone. NMR Biomed.

[13] Gatehouse P.D., Bydder G.M. (2003). Magnetic Resonance Imaging of Short T2 Components in Tissue. Clin Radiol.

[14] Robson M., Benjamin M., Gishen P. (2004). Magnetic resonance imaging of the Achilles tendon using ultrashort TE (UTE) pulse sequences. Clin Radiol.

[15] Weiger M., Pruessmann K.P., Bracher A.-K. (2012). High-resolution ZTE imaging of human teeth. NMR Biomed.

[16] Tesfai A.S., Vollmer A., Özen A.C. (2022). Inductively Coupled Intraoral Flexible Coil for Increased Visibility of Dental Root Canals in Magnetic Resonance Imaging. Invest Radiol.

[17] Lachner S., Utzschneider M., Zaric O. (2021). Compressed sensing and the use of phased array coils in 23Na MRI: a comparison of a SENSE-based and an individually combined multi-channel reconstruction. Z Für Med Phys.

[18] Kurzhunov D., Borowiak R., Reisert M. (2017). 3D CMRO2 mapping in human brain with direct 17O MRI: comparison of conventional and proton-constrained reconstructions. Neuroimage.

[19] Kobayashi N., Goerke U., Wang L. (2015). Gradient-Modulated PETRA MRI. Tomography.

[20] Özen A.C., Novel M.R.I. (2017). Technologies for Structural and Functional Imaging of Tissues with Ultra-short T₂ Values. KIT Scientific Publishing.

[21] Grodzki D.M., Jakob P.M., Heismann B. (2012). Correcting slice selectivity in hard pulse sequences. J Magn Reson.

[22] Li C., Magland J.F., Seifert A.C. (2014). Correction of Excitation Profile in Zero Echo Time (ZTE) Imaging Using Quadratic Phase-Modulated RF Pulse Excitation and Iterative Reconstruction. IEEE Trans Med Imaging.

[23] Breighner R.E., Bogner E.A., Lee S.C. (2019). Evaluation of osseous morphology of the hip using zero echo time magnetic resonance imaging. Am J Sports Med.

[24] Breighner R.E., Endo Y., Konin G.P. (2018). Technical developments: zero echo time imaging of the shoulder: enhanced osseous detail by using MR imaging. Radiology.

[25] Weiger M., Pruessmann K.P. (2019). Short-T2 MRI: Principles and recent advances. Prog Nucl Magn Reson Spectrosc.

[26] Ilbey S., Jungmann P.M., Fischer J. (2022). Single point imaging with radial acquisition and compressed sensing. Magn Reson Med.

[27] Wong S.T.S., Roos M.S. (1994). A strategy for sampling on a sphere applied to 3D selective RF pulse design. Magn Reson Med.

[28] Jackson J.I., Meyer C.H., Nishimura D.G. (1991). Selection of a convolution function for Fourier inversion using gridding (computerised tomography application). IEEE Trans Med Imaging.

[29] Beatty P.J., Nishimura D.G., Pauly J.M. (2005). Rapid gridding reconstruction with a minimal oversampling ratio. IEEE Trans Med Imaging.

[30] Ahmad R., Ding Y., Simonetti O.P. (2015). Edge sharpness assessment by parametric modeling: application to magnetic resonance imaging. Concepts Magn Reson Part A.

[31] Lee K., Sim F.Y. (2021). 3D MRI with CT-like bone contrast–An overview of current approaches and practical clinical implementation. Eur J Radiol.

[32] Wiesinger F., Sacolick L.I., Menini A. (2016). Zero TE MR bone imaging in the head. Magn Reson Med.

[33] Wiesinger F., Bylund M., Yang J. (2018). Zero TE-based pseudo-CT image conversion in the head and its application in PET/MR attenuation correction and MR-guided radiation therapy planning. Magn Reson Med.

[34] Pauly J., Nishimura D., Macovski A. (1969). A k-space analysis of small-tip-angle excitation. J Magn Reson.

