# Supplementary material for: Characterizing Off-center MRI with ZTE
Source: Z Med Phys. 2022 Oct 31;34(3):446–55. doi: 10.1016/j.zemedi.2022.09.002 (PMC11648395; doi:10.1016/j.zemedi.2022.09.002)
Supplement: Supplementary data 1 [file mmc1.docx]

Supporting Information Figures and Table

Off-center MRI with Zero Echo Time (ZTE)

*Serhat Ilbey^1*^, Matthias Jung^2^, Uzay Emir^3,4^, Michael Bock^1^, and Ali Caglar Özen^1^*

*^1^Dept. of Radiology, Medical Physics, Medical Center University of Freiburg, Faculty of Medicine, University of Freiburg, Freiburg, Germany*

*^2^Department of Diagnostic and Interventional Radiology, Medical Center University of Freiburg, Faculty of Medicine, University of Freiburg, Freiburg, Germany*

*^3^Weldon School of Biomedical Engineering, Purdue University*

*^4^School of Health Science Department, Purdue University*

Table S1 MR Imaging Parameters

|  | | **B_0_**  **[T]** | **FOV**  **[mm]** | **Base**  **Resolution** | **Voxel size**  **[mm]** | **TE_1_**  **[ms]** | **TE_2_**  **[ms]** | **TR**  **[ms]** | **α**  **[°]** | **Number**  **of spokes** | $\boldsymbol{\tau}$  **[µs]** | **G**  **[mT/m]** | **Off-center [cm]**  **(LR/AP/SI)** |
| --- | --- | --- | --- | --- | --- | --- | --- | --- | --- | --- | --- | --- | --- |
| **ZTE** | **Phantom** | 1.5 | 180x180x180 | 360x360x360 | 0.5 | 0.05 | N/A | 2 | 3 | 65500 | 8 | 20  30 | 0/0/0  10/0/0 0/-10/0  10/-10/0  10/-10/10 |
|  | **Shoulder** | 1.5 | 180x180x180 | 360x360x360 | 0.5 | 0.05 | 2.8  2.2 | 4.9 4 | 3 | 84000 76000 | 8 | 20  30 | 10/0/0 |
|  | **Knee** | 1.5 | 180x180x180 | 360x360x360 | 0.5 | 0.05 | 2.8  2.2 | 4.9 4 | 6 | 84000 76000 | 8 | 20  30 | 8.5/0/0 |
|  | **Hip** | 3 | 180x180x180 | 360x360x360 | 0.5 | 0.05 | 2.6  2.2 | 4.6 3.8 | 2.9 | 90000 80000 | 8 | 20  30 | 10/3/1 |
|  | | **B_0_**  **[T]** | **FOV**  **[mm]** | **Base**  **Resolution** | **Pixel size**  **[mm]** | **TE**  **[ms]** | **Slice thickness**  **[mm]** | **TR**  **[ms]** | **α**  **[°]** | **Number**  **of Slices** | **BW/pixel** | | **Plane** |
| **TSE** | **Phantom** | 1.5 | 150x150 | 384x384 | 0.4 | 60 | 2 | 5420 | 150 | 28 | 150 | | Axial |
|  | **Shoulder** | 1.5 | 150x150 | 384x384 | 0.4 | 60 | 2 | 5420 | 150 | 28 | 150 | | Coronal |
|  | **Knee** | 1.5 | 150x150 | 384x384 | 0.4 | 60 | 2 | 5420 | 150 | 28 | 150 | | Axial |
|  | **Hip** | 3 | 220x220 | 512x512 | 0.4 | 18 | 2 | 771 | 120 | 34 | 250 | | Axial |


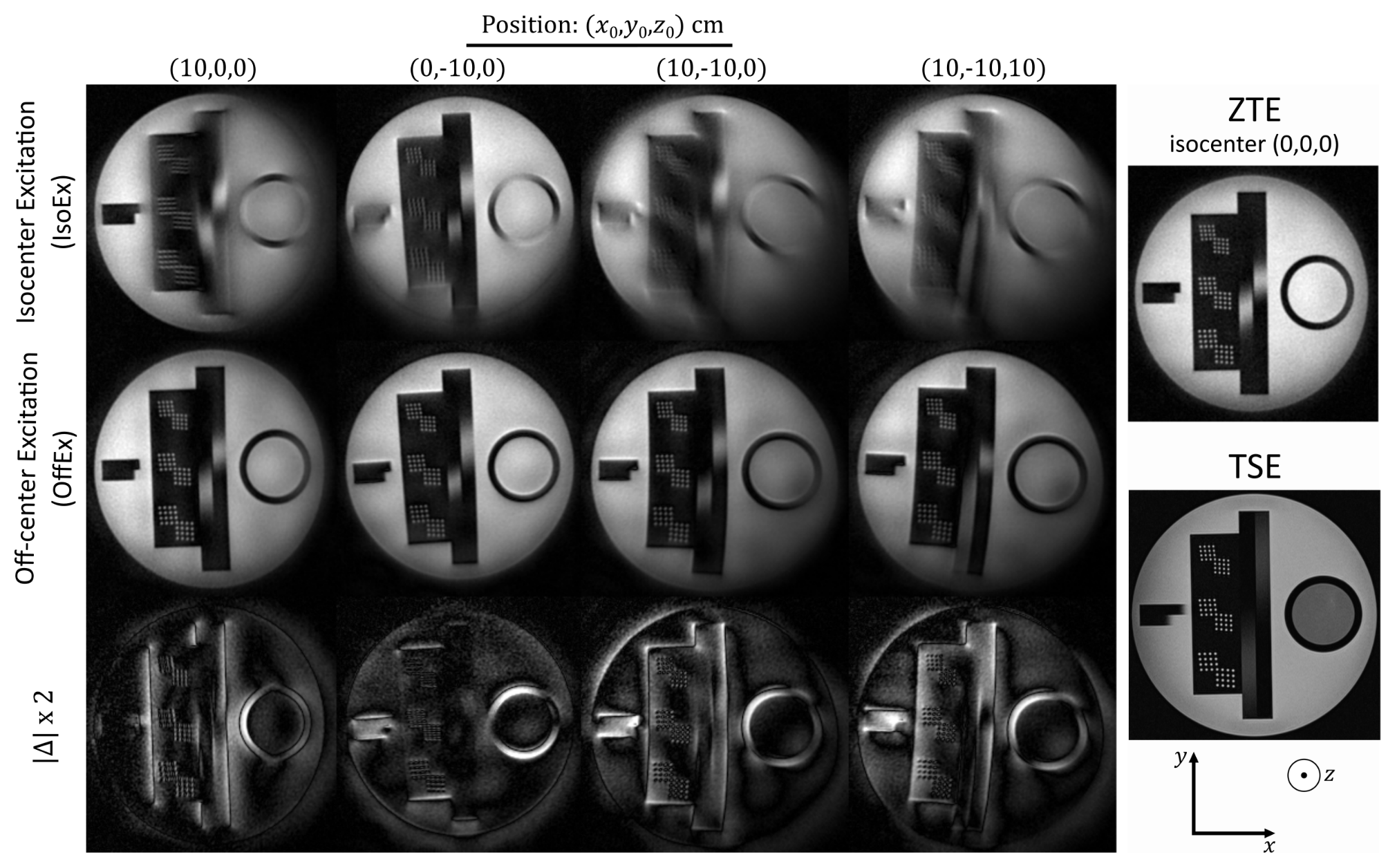


Figure S1 Phantom images acquired with $G$ = 30 mT/m and acquired at isocenter and various off‑centered positions (10,0,0), (0,-10,0), (10,-10,0), and (10,-10,10) using IsoEx and OffEx are shown together with the twice up-scaled absolute difference images ($\left| \Delta\right| x 2$). A TSE image is presented for reference. The severity of the artifacts increased with increasing distance to the isocenter. The OffEx method mitigates the distortion and blurring artifacts at the images of the off-centered objects


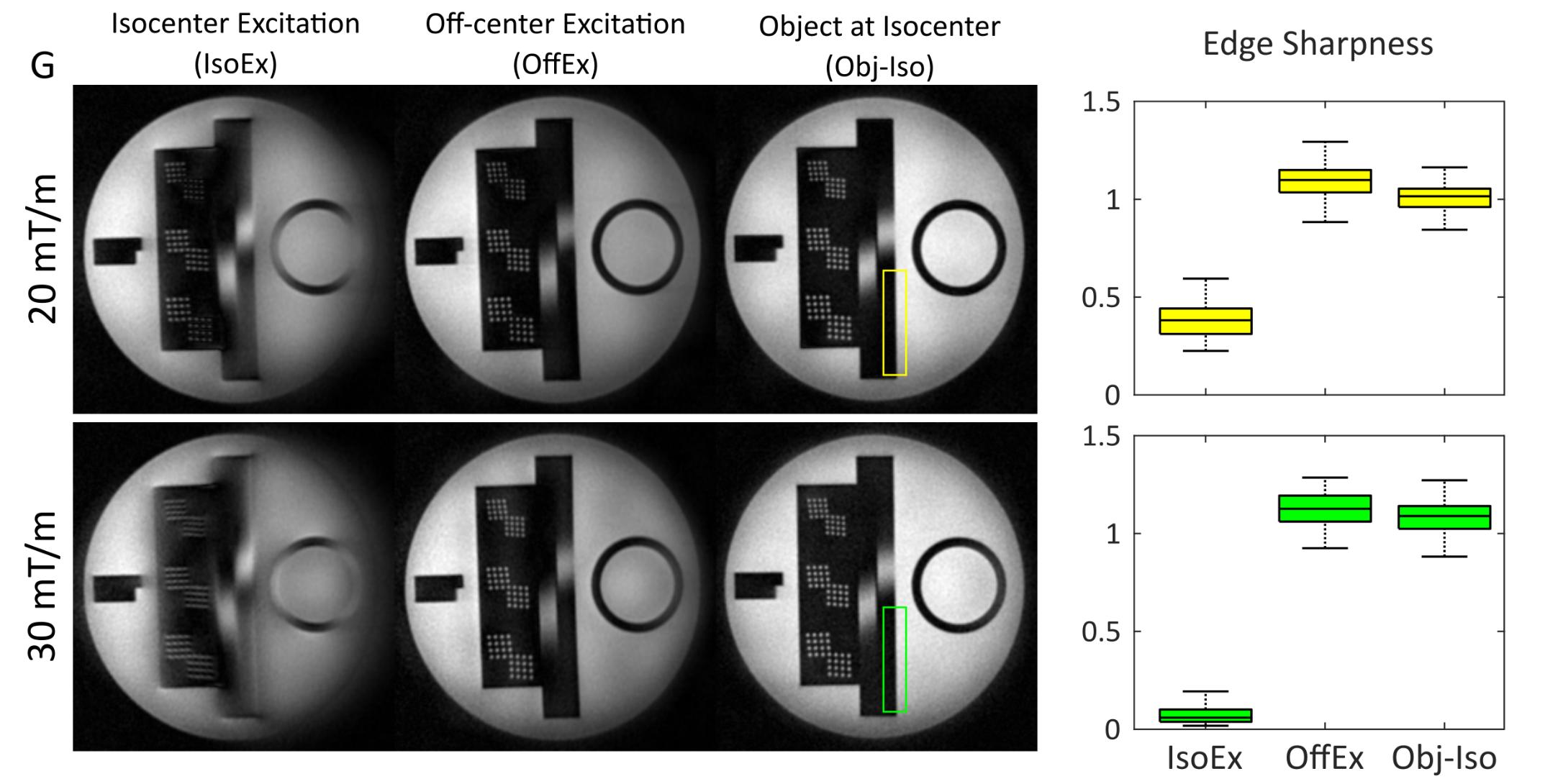


Figure S2 The sharpness ($s$) of the selected edge of the phantom positioned at $\left( x_{0},y_{0},z_{0} \right)$ = (10,0,0) and isocenter. The median of the sharpness of the edges for the object at isocenter (Obj-Iso) and at $\left( x_{0},y_{0},z_{0} \right)$ = (10,0,0) with OffEx and IsoEx acquired with $G$ = 20 mT/m were 1.0, 1.1, and 0.38, respectively. Off-centered phantom acquired with IsoEx had severe blurring artifacts, whereas the edges of the phantom were fully preserved with OffEx. Similar results were obtained for the images acquired with$G$ = 30 mT/m


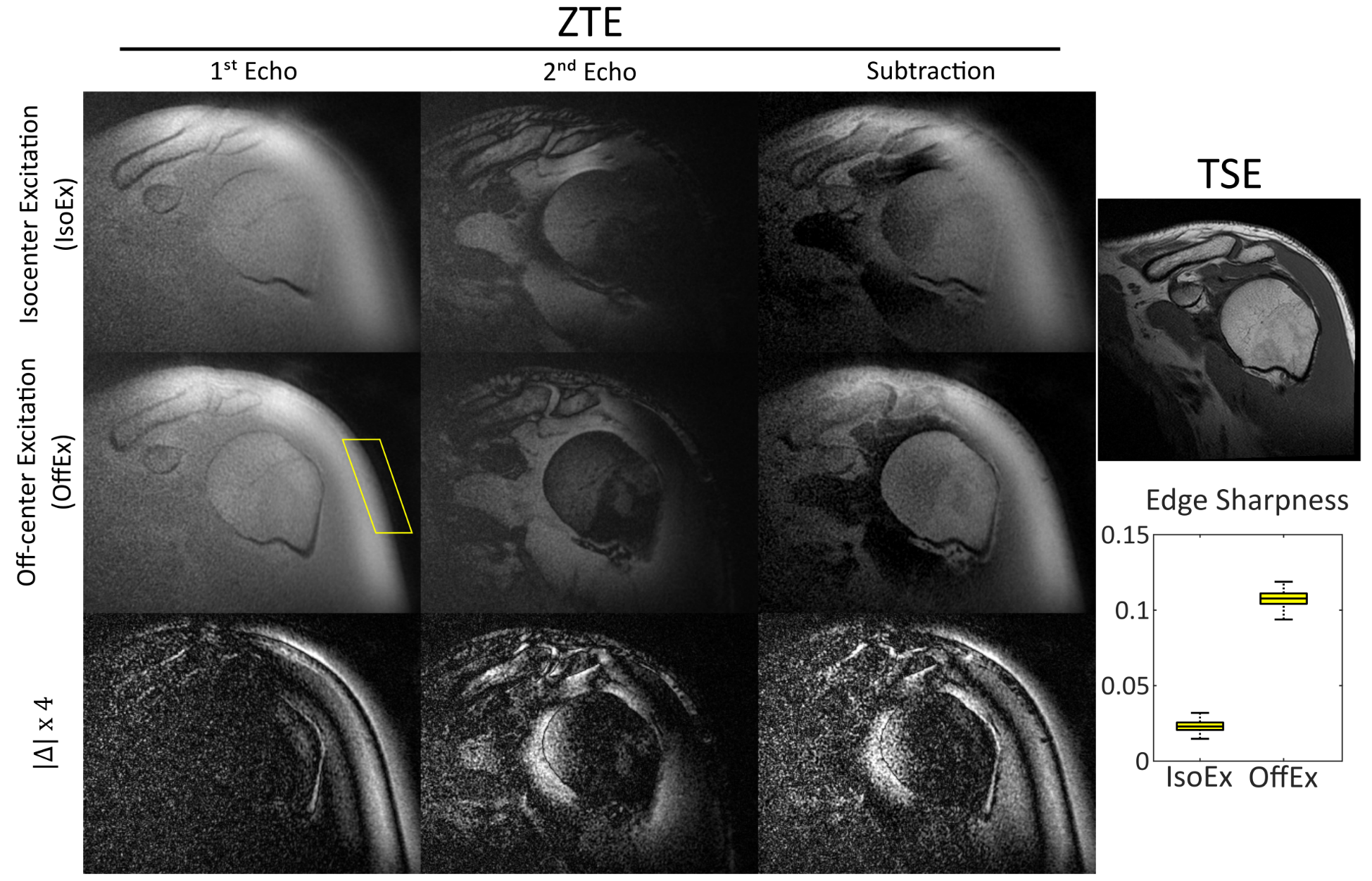


Figure S3 Left shoulder images of the healthy volunteer positioned at $\left( x_{0},y_{0},z_{0} \right)$ = (10,0,0) acquired with double-echo ZTE sequence with $G$ = 30 mT/m using IsoEx and OffEx are presented together with the four times up-scaled absolute difference images ($\left| \Delta\right| x 4$). A conventional T1-weighted TSE image is shown for reference. OffEx mitigates the distortion and blurring artifacts of the images. The sharpness of the selected surrounding fat tissue was increased from 0.02 to 0.11, when OffEx is used. The SNR of the deltoid muscle in the 2^nd^ echo was 0.6 and 5.9 for IsoEx and OffEx, respectively


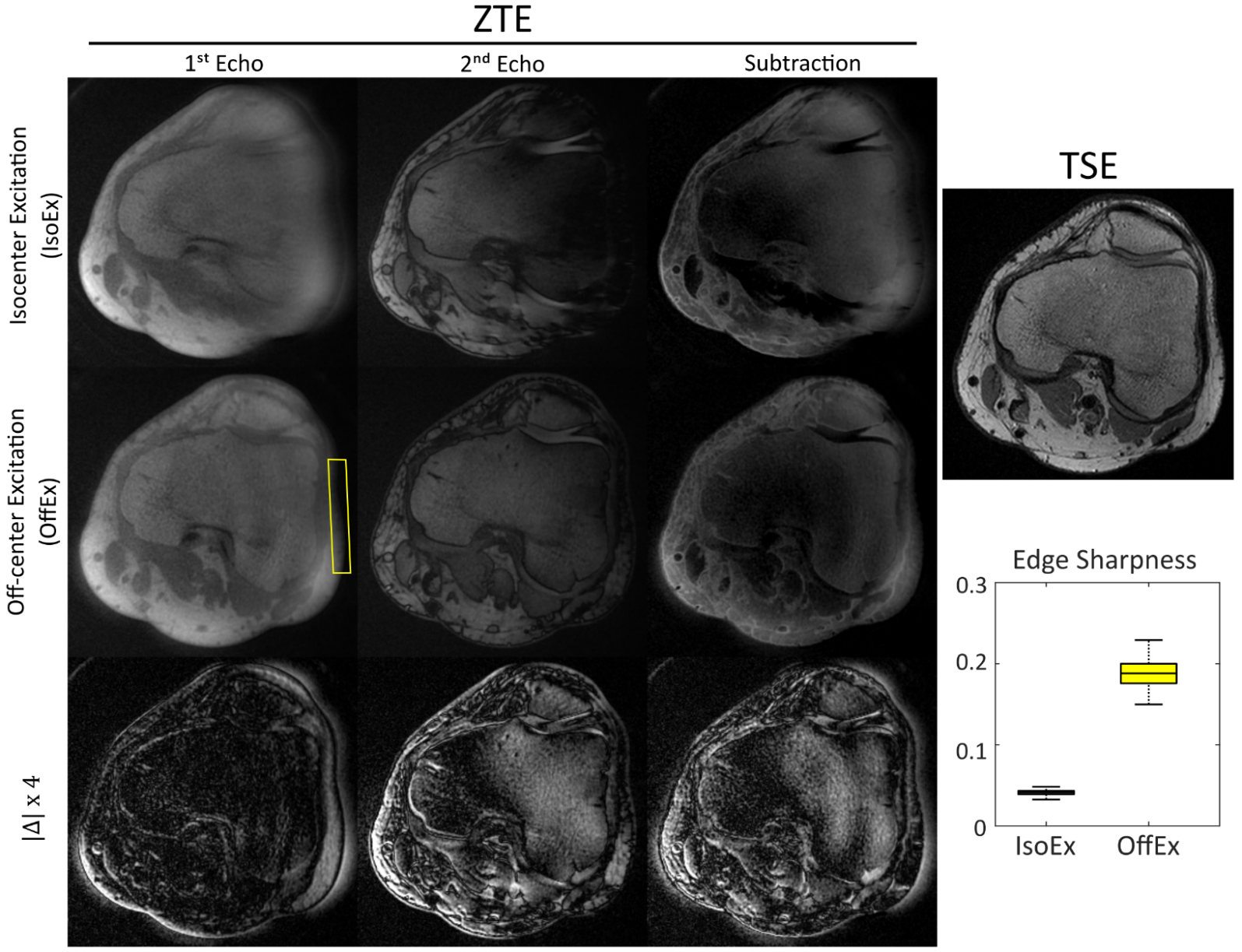


Figure S4 Right knee images of the healthy volunteer positioned at $\left( x_{0},y_{0},z_{0} \right)$ = (8.5,0,0) acquired with double-echo ZTE sequence with $G$ = 30 mT/m using IsoEx and OffEx are presented together with the four times up-scaled absolute difference images ($\left| \Delta\right| x 4$). A conventional T1-weighted TSE image is shown for reference. OffEx mitigates the distortion and blurring artifacts of the images. The sharpness of the selected surrounding fat tissue was increased from 0.04 to 0.18, when OffEx is used. The SNR of the periarticular musculature in the 2^nd^ echo was 30.3 and 50.0 for IsoEx and OffEx, respectively


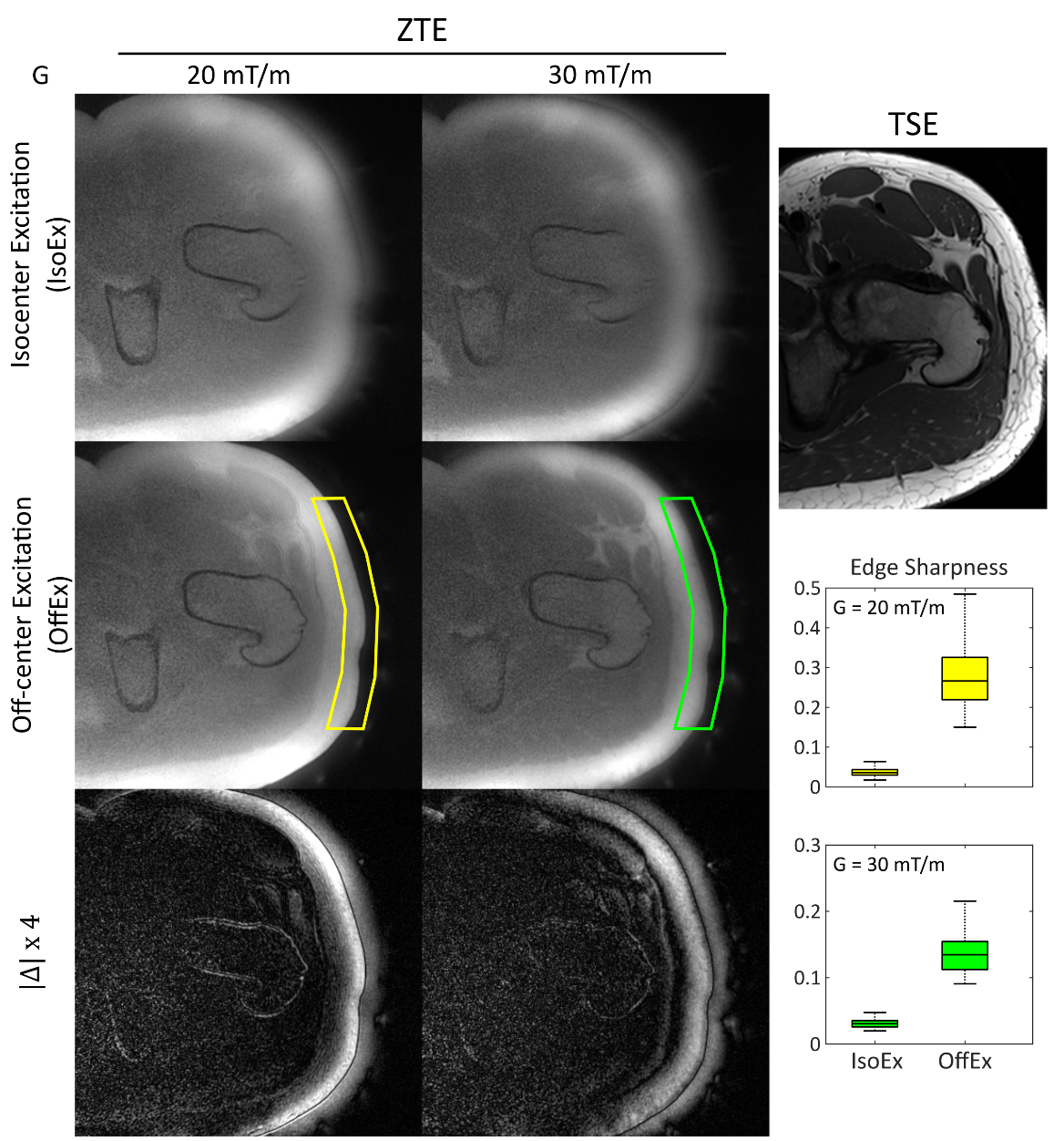


Figure S5 Right hip images of the healthy volunteer positioned at $\left( x_{0},y_{0},z_{0} \right)$ = (10,3,1) acquired with ZTE sequence with $G$ = 20 and 30 mT/m using IsoEx and OffEx are presented together with the four times up-scaled absolute difference images ($\left| \Delta\right| x 4$). In the IsoEx images femur, m. gluteus medius, and the surrounding fat was highly blurry and distorted. For $G$ = 20 mT/m the edge sharpness was increased from 0.04 to 0.27 when OffEx is used, whereas for $G$ = 30 mT/m it was increased from 0.03 to 0.14
