# Supplementary figures and images for: Characterizing Off-center MRI with ZTE
Source: Z Med Phys. 2022 Oct 31;34(3):446–55. doi: 10.1016/j.zemedi.2022.09.002 (PMC11648395; doi:10.1016/j.zemedi.2022.09.002)

## Slide 1
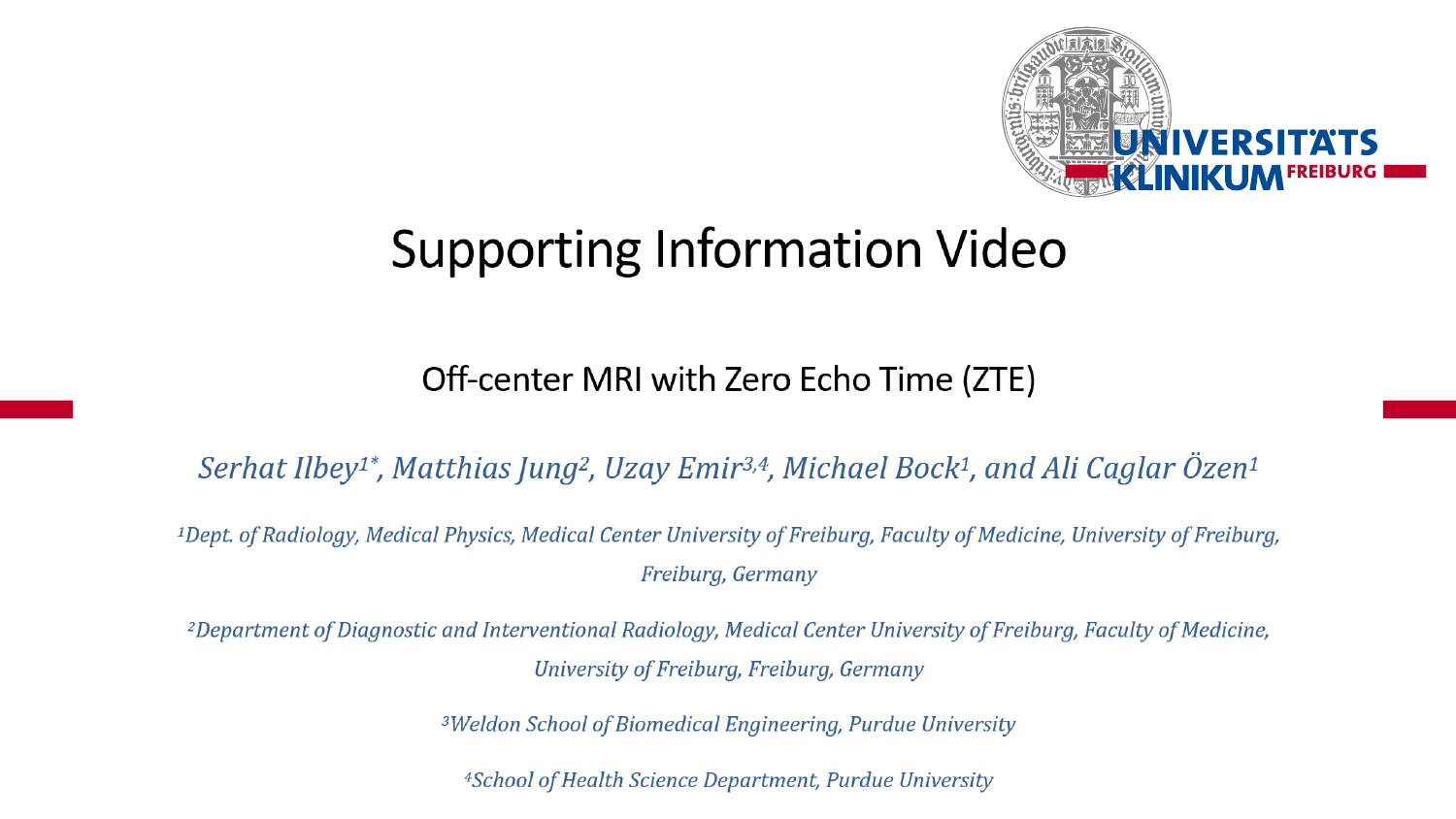

## Slide 2
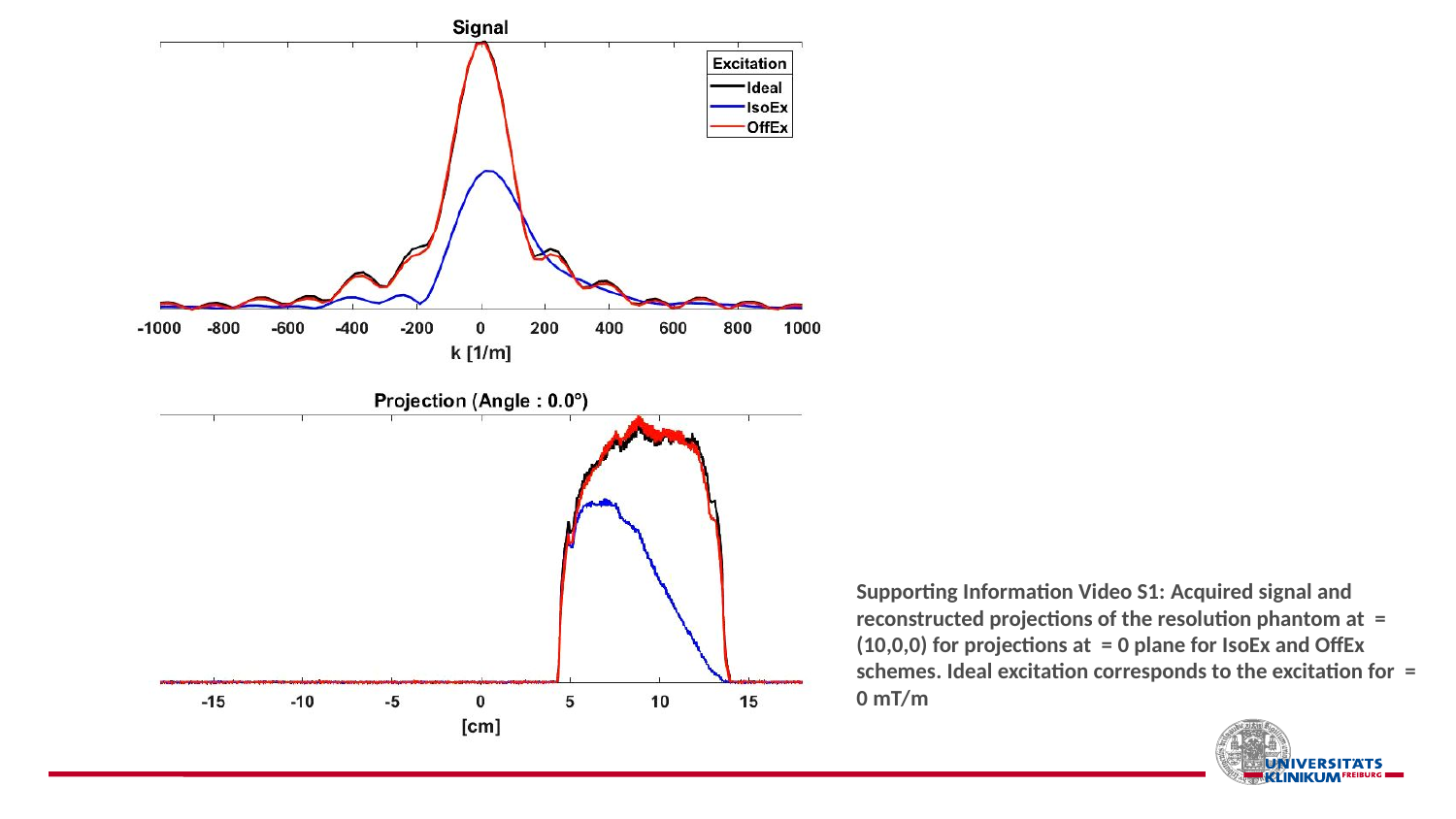

Supplement: Supplementary data 2 [file mmc2.pptx]
